# A multi-omics strategy to understand PASC through the RECOVER cohorts: a paradigm for a systems biology approach to the study of chronic conditions

**DOI:** 10.3389/fsysb.2024.1422384

**Published:** 2025-01-07

**Authors:** Jun Sun, Masanori Aikawa, Hassan Ashktorab, Noam D. Beckmann, Michael L. Enger, Joaquin M. Espinosa, Xiaowu Gai, Benjamin D. Horne, Paul Keim, Jessica Lasky-Su, Rebecca Letts, Cheryl L. Maier, Meisha Mandal, Lauren Nichols, Nadia R. Roan, Mark W. Russell, Jacqueline Rutter, George R. Saade, Kumar Sharma, Stephanie Shiau, Stephen N. Thibodeau, Samuel Yang, Lucio Miele, NIH Researching COVID to Enhance Recovery (RECOVER) Consortium

**Affiliations:** ^1^ Department of Medicine, Division of Gastroenterology and Hepatology, University of Illinois Chicago, Chicago, IL, United States; ^2^ Cardiovascular Division and Channing Division of Network Medicine, Department of Medicine, Brigham and Women’s Hospital and Harvard Medical School, Boston, MA, United States; ^3^ Department of Medicine, Howard University, Washington, DC, United States; ^4^ Department of Medicine, Division of Data Driven and Digital Medicine (D3M), New York, NY, United States; ^5^ Charles Bronfman Institute for Personalized Medicine, Mount Sinai Clinical Intelligence Center, Icahn School of Medicine at Mount Sinai, New York, NY, United States; ^6^ RTI International, Durham, NC, United States; ^7^ Linda Crnic Institute for Down Syndrome, University of Colorado Anschutz Medical Campus, Aurora, CO, United States; ^8^ Department of Pathology and Laboratory Medicine, Children’s Hospital Los Angeles, Los Angeles, CA, United States; ^9^ Department of Pathology, Keck School of Medicine, University of Southern California, Los Angeles, CA, United States; ^10^ Intermountain Medical Center Heart Institute, Murray, UT, United States; ^11^ Department of Medicine, Division of Cardiovascular Medicine, Stanford University, Stanford, CA, United States; ^12^ Department of Biology, Northern Arizona University, Flagstaff, AZ, United States; ^13^ Pathogens Genomics Program, Translational Genomics Institute (TGen), Phoenix, AZ, United States; ^14^ Department of Biology, University of Oxford, Oxford, United Kingdom; ^15^ Channing Department of Network Medicine, Brigham and Women’s Hospital, Harvard University, Boston, MA, United States; ^16^ RECOVER patient representative, Durham, NC, United States; ^17^ Department of Pathology and Laboratory Medicine, Emory University School of Medicine, Atlanta, GA, United States; ^18^ Gladstone Institute of Virology, Gladstone Institutes, San Francisco, CA, United States; ^19^ Department of Urology, University of California San Francisco, San Francisco, CA, United States; ^20^ Department of Pediatrics, Division of Pediatric Cardiology, University of Michigan, Ann Arbor, MI, United States; ^21^ Department of Obstetrics and Gynecology, University of Texas Medical Branch, Galveston, TX, United States; ^22^ Department of Obstetrics and Gynecology, Eastern Virginia Medical School, Norfolk, VA, United States; ^23^ Center for Precision Medicine, University of Texas San Antonio Health Sciences Center, San Antonio, TX, United States; ^24^ Department of Medicine, Division of Nephrology, University of Texas San Antonio Health Sciences Center, San Antonio, TX, United States; ^25^ Department of Biostatistics and Epidemiology, Rutgers School of Public Health, Piscataway, NJ, United States; ^26^ Department of Laboratory Medicine and Pathology, Mayo Clinic, Rochester, MN, United States; ^27^ Department of Emergency Medicine, Stanford University, Stanford, CA, United States; ^28^ Department of Genetics, School of Medicine, Louisiana State University Health Sciences, Center New Orleans, New Orleans, LA, United States

**Keywords:** COVID-19, PASC, RECOVER, systems biology, multi-omics

## Abstract

Post-Acute Sequelae of SARS-CoV-2 infection (PASC or “Long COVID”), includes numerous chronic conditions associated with widespread morbidity and rising healthcare costs. PASC has highly variable clinical presentations, and likely includes multiple molecular subtypes, but it remains poorly understood from a molecular and mechanistic standpoint. This hampers the development of rationally targeted therapeutic strategies. The NIH-sponsored “Researching COVID to Enhance Recovery” (RECOVER) initiative includes several retrospective/prospective observational cohort studies enrolling adult, pregnant adult and pediatric patients respectively. RECOVER formed an “OMICS” multidisciplinary task force, including clinicians, pathologists, laboratory scientists and data scientists, charged with developing recommendations to apply cutting-edge system biology technologies to achieve the goals of RECOVER. The task force met biweekly over 14 months, to evaluate published evidence, examine the possible contribution of each “omics” technique to the study of PASC and develop study design recommendations. The OMICS task force recommended an integrated, longitudinal, simultaneous systems biology study of participant biospecimens on the entire RECOVER cohorts through centralized laboratories, as opposed to multiple smaller studies using one or few analytical techniques. The resulting multi-dimensional molecular dataset should be correlated with the deep clinical phenotyping performed through RECOVER, as well as with information on demographics, comorbidities, social determinants of health, the exposome and lifestyle factors that may contribute to the clinical presentations of PASC. This approach will minimize lab-to-lab technical variability, maximize sample size for class discovery, and enable the incorporation of as many relevant variables as possible into statistical models. Many of our recommendations have already been considered by the NIH through the peer-review process, resulting in the creation of a systems biology panel that is currently designing the studies we proposed. This system biology strategy, coupled with modern data science approaches, will dramatically improve our prospects for accurate disease subtype identification, biomarker discovery and therapeutic target identification for precision treatment. The resulting dataset should be made available to the scientific community for secondary analyses. Analogous system biology approaches should be built into the study designs of large observational studies whenever possible.

## 1 Introduction

The term Post-Acute Sequelae of SARS-CoV-2 infection (PASC), also known as “Long COVID”, refers to numerous conditions associated with widespread morbidity and rising healthcare costs. PASC has highly variable clinical presentations, and likely includes multiple molecular subtypes (Thompson et al., 2023; Sherif et al., 2023). The NIH-sponsored “Researching COVID to Enhance Recovery” (RECOVER) initiative includes retrospective/prospective cohort studies including an adult cohort (Horwitz et al., 2023), a cohort of pregnant adults (Metz et al., 2023; Reel et al., 2021) and a pediatric cohort (Gross et al., 2024; Reel et al., 2021). These studies aim to enroll a total of 12,580 adult non-pregnant patients, 2,300 adult pregnant patients and 19,300 pediatric patients to rapidly improve our understanding of and ability to predict, treat, and prevent Post-Acute Sequelae of SARS-CoV-2 infection (PASC, or “Long COVID”) through deep clinical phenotyping and laboratory studies. THE RECOVER “OMICS” Task Force was charged with developing recommendations based on published evidence and the experiences of its members, to incorporate multi-omics into the analysis of RECOVER results.

## 2 Methods

### 2.1 Objectives

The “OMICS” task force of the RECOVER study, a multi-disciplinary committee including clinicians, pathologists, laboratory scientists and data scientists, was charged with developing recommendations to apply cutting-edge system biology technologies to achieve the goals of RECOVER. The task force met biweekly over 14 months, to evaluate published evidence, examine the possible contribution of each “omics” technique to the study of PASC, as well as the potential limitations of each technique, and develop a consensus recommendation. The work was divided into two stages. During the first stage, sub-committees with specific expertise on an “omics” technique examined evidence supporting the use of that technique to study PASC, the type of data it could generate and the mechanistic questions it could answer, based on published evidence and the experiences of its members, to incorporate multi-omics into the analysis of RECOVER results. Each sub-committee presented to the entire task force. During the second stage, the task force combined the findings of each sub-committee into a comprehensive study design recommendation.

## 3 Results and discussion

The OMICS task force recommended that integrated, longitudinal, simultaneous multi-omics studies of participant biospecimens be performed on the entire RECOVER cohort through centralized laboratories, as opposed to multiple smaller studies using one or few analytical techniques.

The RECOVER adult protocol (Horwitz et al., 2023) includes multiple biospecimen collections: nasopharyngeal or nasal swab, 2 8.5 mL aliquots of blood in serum separation tubes, 4 × 8 ml aliquots of blood in cell preparation tubes, 2 × 2.7 mL aliquots of blood in sodium citrate tubes for plasma proteomics, 1 × 10 ml aliquot of blood in EDTA tube, 1 2.5 mL aliquot of blood in PAXgene RNA tube, 1 × 10 ml urine (no additives), 1 × 2mL aliquot of saliva in Oragene OGR 600 and 1 25 mL aliquot of stool. Of these, stool is sent by participants while the other samples are processed locally as per protocol specifications and shipped in batches to the central tissue bank. Participants who consent to biospecimen donation for future research are asked to provide blood and nasopharyngeal/nasal swab biospecimens at enrollment, 90 and 180 days after the index date (date of first infection or negative COVID test), and then annually (Horwitz et al., 2023). Saliva is collected upon enrollment for genetic analysis. Urine and stool are collected biannually. Additionally, a battery of clinical laboratory tests is performed in CLIA-certified laboratories at enrollment, 90 and 189 days after the index date, and thereafter, abnormal tests are repeated annually. Specific symptoms or abnormal study results trigger “Tier 2” or “Tier 3” assessments (see (Horwitz et al., 2023) for details). A SARS-CoV-2 PCR test is performed at enrollment for all “uninfected” participants, who are also tested for SARS-CoV-2 nucleocapsid antibodies spike protein antibodies for unvaccinated participants.

In addition to study visits, imaging and laboratory tests, participants complete multiple surveys, using validated survey instruments whenever possible, at 90-day intervals throughout the study. At enrollment, data are collected on demographics, social determinants of health (SDOH), disability, characteristics of the initial SARS-CoV-2 infection (if applicable), pregnancy (if applicable), vaccination status, comorbidities, medications, and PASC symptoms. Subsequently, at 90-day intervals, data are collected on interim infections, time-varying social determinants, vaccinations, comorbidities, medications and symptoms (Horwitz et al., 2023). The PASC symptom survey was developed for RECOVER and includes an overall quality of life instrument (PROMIS-10) and screening for core symptoms (43 for biological males and 46 for biological females) drawn from existing literature plus input from patient representatives and investigators. Questions about depression, anxiety, post-traumatic stress disorder (PTSD), and grief are also included. Report of a symptom may trigger additional questions about that symptom. Details of survey instruments are in the original reference (Horwitz et al., 2023).

The pregnancy study (Metz et al., 2023) follows a similar design to the non-pregnant adult study, enrolling participants with suspected, probable or confirmed SARS-CoV-2 infection during pregnancy, or documented lack of exposure to SARS-CoV-2 during pregnancy. Study procedures and biospecimen collections are analogous to those in the non-pregnant adult study (Metz et al., 2023), with modifications for breastfeeding or *postpartum* participants, and additional health and developmental assessments for babies exposed *in utero* to SARS-CoV-2.

The RECOVER pediatric study (Gross et al., 2024) has a similar design, with limitations due to the age range of participants. All pediatric participants complete a single Tier 1 visit including PROMIS global health measures and symptom screening. This visit includes a donation of saliva and capillary blood. Depending on infection status, clinical history, symptoms and probability of PASC, pediatric participants are promoted to Tier 2 or Tier 3, which include additional biospecimen donations during the acute and post-acute phase of PASC, as well as additional clinical assessments and surveys. The types, aliquot numbers, and cadence of biospecimen collections are described in detail in (Gross et al., 2024).

In summary, each RECOVER study will generate vast longitudinal datasets including clinical, demographic, medication, SDOH and lifestyle data for each participant, as well as sufficient types and numbers of biospecimen aliquots to permit a comprehensive, longitudinal multi-omics investigation. Potential environmental exposures can furthermore be estimated from census tract or ZIP code data.

The multi-dimensional molecular dataset generated by the multi-omics investigation should be correlated with the deep clinical phenotyping performed through RECOVER, as well as with information on demographics, comorbidities, social determinants of health, the exposome and lifestyle factors collected through RECOVER surveys, that may contribute to the clinical presentations of PASC. Data generation and analytical strategies should leverage integrative bioinformatics and machine learning.

A major advantage, and a potential challenge, of multi-omics approaches is that datasets derived from different analytical techniques and measured using different scales must be integrated. Approaches including multi-omics integration paired with ML have been gaining popularity in clinical and biomedical research (see (Reel et al., 2021; Niranjan et al., 2023)), though this field is rapidly evolving. An important advantage inherent in multi-dimensional measurements is that the extent to which different measurements agree with each other or not is potentially informative. For instance, transcriptomic data may or may not be reflected in the relative abundance of protein products, or quantitative differences in non-coding RNA expression may or may not translate into relative abundance of potential target mRNAs, the proteins they encode or the metabolites that these proteins may process. System biology approaches based on multi-omics have been used successfully in the study of cardiovascular disease (Joshi et al., 2021).

With respect to PASC, strategies similar to what we propose have been used on a smaller scale. ML has been used in the context of a multi-step analytical strategy to combine proteomic and metabolomic data to generate a multi-omics biomarker predictive of the risk of PASC (Wang et al., 2023) and give insights on the metabolic pathways altered during PASC. Dimensionality reduction was achieved through unsupervised cluster analysis followed by autoencoder (AE), using a three-layer neural network. Supervised ML was then used to identify the minimal number of molecules predictive of adverse clinical outcomes. This study, though very promising, was limited by small sample size (117, of whom 105 were used as a training cohort for model development and 10% as a validation cohort), the severity of acute COVIDs in the patients enrolled and the absence of a vaccinated group. Despite these limitations, these results indicate that similar analytical strategies can be used successfully on a much larger sample with broader phenotyping, to discover predictive biomarkers, therapeutic targets and risk factors and to generate mechanistic hypotheses.

Within the RECOVER study, an unsupervised ML approach has been used to identify clinical subtypes of PASC, after symptoms differentiating infected from uninfected patients were identified using LASSO (least absolute shrinkage and selection operator) (Thaweethai et al., 2023).

**FIGURE 1:**
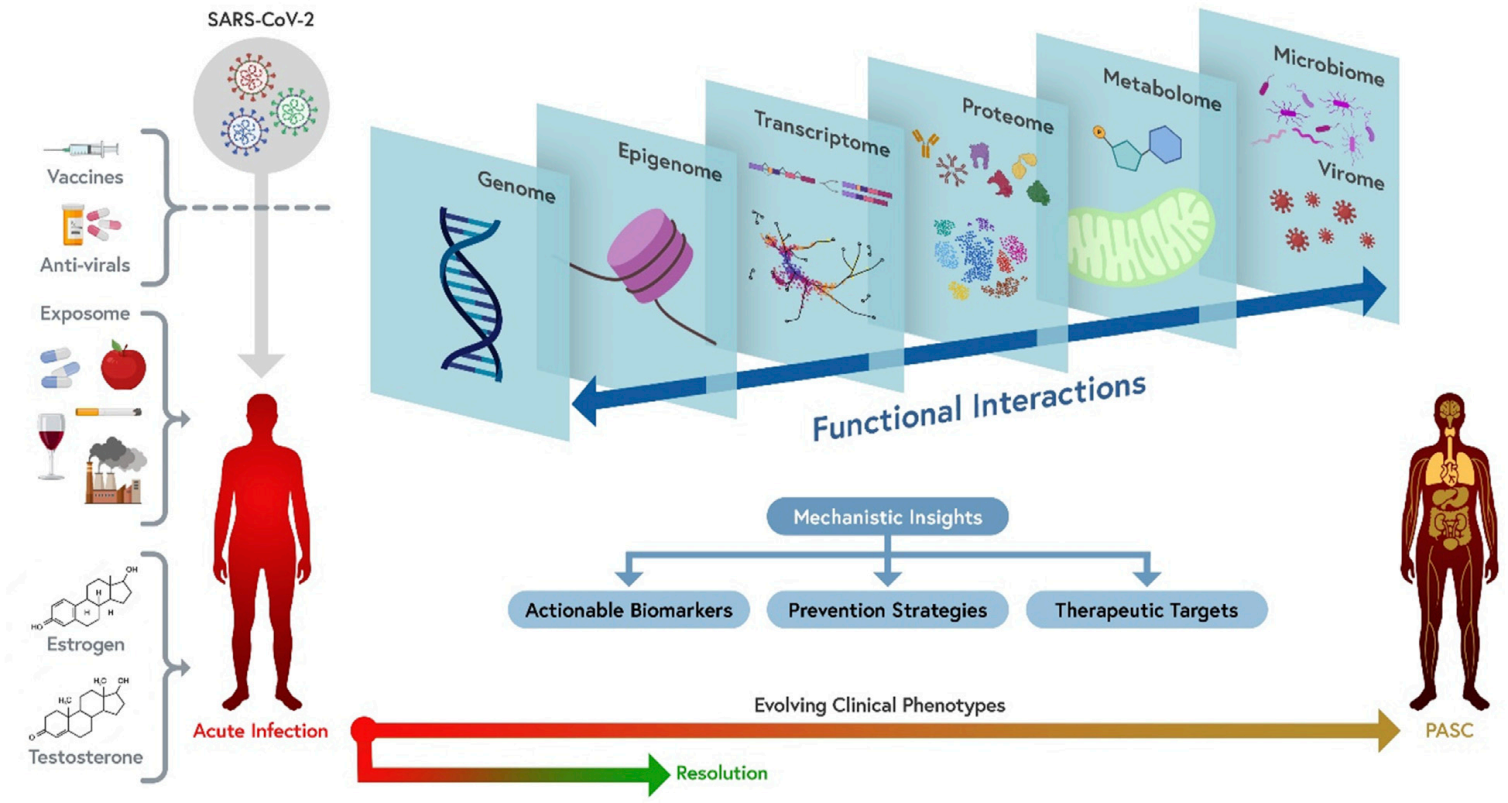
A comprehensive multi-omics approach to the mechanism(s) of PASC. From left to right: PASC is a consequence of infection with SARS-CoV-2. Different viral variants or sub-variants (represented in different colors) may have different probability of causing PASC or be associated with different presentations (e.g., due to different ability to cause persistent infection, to trigger pathogenic antibody responses, or to damage vascular endothelium). Vaccines and anti-viral agents can decrease the risk of PASC by interfering with viral persistence and replication. Multiple exposures, including diet, medications, tobacco, alcohol, environmental pollutants and co-morbidities, socioeconomic and psychosocial exposures, as well as sex hormones, can potentially affect the risk and clinical presentations of PASC. The combined effect of these factors results into evolving clinical phenotypes ranging from acute COVID-19 resolution to PASC through a number of mechanisms that can be best understood by simultaneously interrogating the multi-omics landscape of patients, including individual genomics, epigenomics, bulk and single-cell transcriptomics, plasma and cellular proteomics, metabolomics, and microbiome/virome. These different dimensions functionally interact with one another to determine pathogenetic mechanisms (e.g., persistent viral infection, modulated by individual genetics, triggers immune, inflammatory and metabolic changes that are in turn modulated by the intestinal and respiratory microbiomes and potentially by reactivation of other viruses). Insights generated by an integrated multi-omics investigation of patients with well-characterized clinical phenotypes are likely to identify actionable biomarkers (which may discriminate between PASC molecular subtypes), as well as therapeutic targets and prevention strategies. Orthogonal multi-omics tests repeated over time are the most informative approach to capture the pathogenesis of the different clinical presentations of PASC and their evolution over time.

Highly multiplexed “omics” approaches measure common clinical analytes and many more parameters (Table 1) at a fraction of the cost of traditional clinical tests, oftentimes using similar quantities of specimens (Table 2). In a multi-omics approach, analytes within each category (e.g., proteins, lipids, nucleic acids, metabolites, and microbes) are all measured simultaneously, generating high-content data that is more than the sum of its parts. This approach allows the discovery of new molecular signatures to enhance our understanding of complex disease pathophysiology. These signatures may occur within a single analyte category, but more likely cover more complex patterns that span multiple molecular layers, e.g., genomics, epigenomics, transcriptomics, proteomics, lipidomics, metabolomics, and microbiomics (Figure 1). Deep multi-omics profiling will allow us to explore a broad spectrum of pathophysiological mechanisms (Table 3), define gene-environment interactions involved in the pathogenesis of PASC, identify molecular subtypes and candidate biomarkers and propose mechanism-based therapeutic strategies. The relative contributions of each “omics” we evaluated and considerations on data generation and analysis are described below.

**TABLE 1:** Complementary data types captured by multi-omics assays.

Data type	Assays							
	SNP NGS	Epigenome	Bulk RNASeq	scRNASeq	Proteome	CyTOF	Metabolome	Microbiome
Genetic risk factors	+							
Epigenetic modifications		+	+					
mRNA/splice variants			+					
ncRNA			+					
Viral RNA			+					
Immune phenotyping			+	+	+	+		+
Antibodies					+			
Cytokines, chemokines					+			
Peptide hormones					+			
Coagulation factors					+			
Viral proteins					+			
Post-translational modifications					+	+		
Human Metabolites							+	
Bacterial metabolites							+	
Toxins/drugs							+	
Vitamins/hormones							+	
Bacterial diversity								+

**TABLE 2:** Approximate sample requirements for multi-omics assays.

Approximate amount of material	Assays							
	SNP NGS, GWAS	Methylome	Bulk RNASeq	scRNASeq	Proteome	CyTOF	Metabolome	Microbiome
	300–400 ng DNA (50 µL blood)	50–100 ng DNA	PBMC in 1 mL blood (250 ng RNA)	100,000 PBMC (20–30 µL blood)	400–500 µL EDTA plasma	106 cells (200–300 µL blood)	50–100 µL plasma	<500 mg stool

**TABLE 3:** Multi-omics assays generate information relevant to testing multiple mechanistic hypotheses for PASC.

Pathogenetic hypothesis	Assays							
	SNP lp-NGS, GWAS	Epigenome	Bulk RNASeq	scRNASeq	Proteome	CyTOF	Metabolome	Microbiome
Genetic predisposition	**+**							
Viral persistence			**+**		**+**			
Intra-patient viral evolution			**+**					
Non-SARS-CoV2 viral reactivation (EBV, others)			**+**		**+**			
Autoimmunity	**+**	**+**	**+**	**+**		**+**		**+**
Chronic inflammation		**+**	**+**	**+**	**+**	**+**	**+**	**+**
Endothelial damage					**+**	**+**		
Coagulation abnormalities					**+**			
Dysbiosis							**+**	**+**
Chronic stress		**+**	**+**	**+**	**+**		**+**	
Endocrine dysfunction					**+**		**+**	
Toxic exposures		**+**			**+**		**+**	

### 3.1 Evidence supporting multi-omics technologies used in COVID-19 and PASC studies

#### 3.1.1 Genomics

Genomics is an invaluable asset to understand disease risk, mechanism and etiology, and to serve as a backbone to allow for better modeling of multi-omics profiles in patient populations. Several genome-wide association studies (GWAS) have identified reproducible associations between specific loci and risk and outcomes of acute COVID-19 (Ferreira et al., 2022) with the most reproducible being with LZTFL1 and contiguous regions on 3q21.31 and ABO on 9q34.2. A recent GWAS study, currently in pre-print, detected an association between a locus near the FOXP4 gene and risk of developing PASC (Lammi et al., 2023). That study analyzed data from 24 studies conducted in 16 countries, totaling 6,450 PASC cases and 1,093,995 controls. However, most of the patients were of European ancestry, and this study should be replicated in a more diverse cohort. In GWAS studies, sample size and composition of study population (e.g., case/control ratio, ancestry, genetic admixture, *etc.*) are critical. FOXP4 is a broadly expressed transcription factor. Lammi et al. (Lammi et al., 2023) analyzed single-cell RNASeq data to confirm the expression of FOXP4 in surfactant-producing Type II alveolar cells and granulocytes. This correlation supports a possible mechanistic link, and demonstrates the importance of integrative multi-omics approaches. A recent computational study analyzed the evolution of predicted CD8 T-cell epitopes in SARS-CoV-2 variants and its correlation with clinical outcomes of acute COVID-19 in patients with different HLA genotypes, illustrating the importance of integrated analysis of viral and patient genomic data with clinical data (Kim et al., 2024). A similar approach could be used with PASC, and/or PASC clinical subtype, as an outcome. Beyond GWAS or other genetic analyses, genotyping data can be used in conjunction with other multi-omics profiles to increase the likelihood of discovery. Identification of molecular quantitative trait loci (QTL) can be used to identify possible pathogenetic pathways (Debnath et al., 2020). Different technologies can be used to obtain genotyping information in PASC cases: high-density SNP-chips or low-pass sequencing are established platforms. Emerging technologies, such as nanopore long-read sequencing (Cuppen et al., 2022; Pervez et al., 2022), may also reduce the cost of whole-genome and whole-transcriptome sequencing.

#### 3.1.2 Epigenomics

Epigenomics measure molecular events that regulate chromatin accessibility and expression, which can reflect long-term physiological states. Using methods such as chromatin immunoprecipitation sequencing (ChIP-seq), CUT&RUN, or assay for transposase-accessible chromatin using sequencing (ATAC-seq) (Yan et al., 2020; Sun et al., 2019), which can also be used in single-cell applications (Kashima et al., 2020), many epigenetic processes have been identified and associated with complex traits. One of the best characterized is DNA methylation (5 methyl-cytosine), which is altered in numerous human diseases. There is compelling evidence that changes in DNA methylation profiles are detectable in viral infections such as HIV (Bednarik et al., 1990; Zhang et al., 2016) and MERS (Menachery et al., 2018). In an epigenome-wide association study (EWAS) (Bhat and Jones, 2022), DNA methylation differences associated with a phenotype can be assessed at hundreds of thousands of cytosine-phosphate-guanine (CpG) sites across the epigenome. Several EWAS of COVID-19 in the literature found distinct patterns of DNA methylation associated with disease severity early in the disease course (Castro de Moura et al., 2021; Corley et al., 2021; Balnis et al., 2021; Zhou et al., 2021). EWAS in the ongoing Norwegian Corona Cohort Study also assessed whether there were differentially methylated CpGs between those with PASC (N = 41) compared to a remission group (N = 63), but did not find significant differences. However, the study was not longitudinal, and the authors point out that their sample size for PASC was small (Lee et al., 2022). The same study identified 3 differentially methylated sites associated with acute COVID-19 severity, including hypomethylation of IFI44L, an interferon response gene also associated with COVID-19 severity (Castro de Moura et al., 2021).

#### 3.1.3 Transcriptomics

a) **Bulk Transcriptomics:** RNA transcripts act as intermediary components between genetic information and protein synthesis, and carry specific functions themselves. Transcripts are a regulation hub that responds to both environmental and genetic control, thus playing a major role in the molecular characterization of diseases. Non-coding RNAs fine-tune the expression levels of coding RNAs and their protein products, providing an additional level of regulation. Given its role as an ‘integration hub’ between genetic variation and environmental exposures, the transcriptome dataset is a key layer in multi-omics approaches. Bulk RNA sequencing (RNASeq) can measure the relative abundance of individual transcripts, and determine differences in mRNA splicing isoforms and RNA editing. Whole blood transcriptome analysis can accurately measure the expression levels of >16,000-20,000 RNA species, both protein-coding and non-coding, thus providing one of the most high-quality and high-content multi-omics datasets. Bulk transcriptomics integrates the effects of multiple key variables that can dynamically affect gene expression in blood cells (e.g., metabolic state, epigenetic variation, exposure to medications, stress *etc.*). Transcriptional signatures in the blood or cells of COVID-19 patients can help identify causal factors for acute or chronic complications as well as potential therapeutic targets (Jha et al., 2020; Asano et al., 2022). Recently, a long non-coding RNA-based ML model has been used to identify an RNA (LEF1-AS1) predictive of acute COVID-19 mortality in a ML-driven study of 1,286 patients in 15 institutions (Devaux et al., 2024). Additionally, a candidate signature of acute COVID-19 including 3 long non-coding RNA, 2 cytokines and 2 proteins in peripheral blood mononuclear cells (PMBCs) has been identified using a ML approach (Heydari et al., 2024). This study had a fairly small sample size (28 COVID-19 patients and 17 controls), but it illustrates the promise of multi-analyte biomarkers including RNAs in COVID-19. Current bioinformatics deconvolution approaches enable effective estimation of cell-type fraction and cell type specific gene expression in the peripheral immune system from bulk transcriptome data, offering a powerful tool for immune-phenotyping (Chen et al., 2018) that is complementary to plasma and cellular proteomics. Bulk samples employed for RNAseq can also be used for in-depth immune repertoire analyses (Galson et al., 2020). These data also allow prediction of physiological states, such as PANoptosis (Yang et al., 2024; Dai et al., 2023) or innate immunity activation (Karki and Kanneganti, 2022), and upstream regulators of these states (e.g., transcription factors, protein kinases, hormones), thus enabling the identification of potential therapeutic targets. Critically, transcriptomic data can also allow for the identification of circulating SARS-CoV-2 viral load from whole blood (including viral variant calling and, given sufficient sequence coverage, detection of intra-patient viral evolution), thus constituting an important tool to assess persistent viremia from sources such as vascular beds. Further virome/microbiome analyses of these data can capture other viruses/bacteria that may contribute to PASC pathophysiology (e.g., EBV). Several such transcriptome analyses have been completed for acute COVID-19 and PASC (Thompson et al., 2023; Hadjadj et al., 2020; Lucas et al., 2020; Sposito et al., 2021; Sullivan et al., 2021; Ziegler et al., 2021; Galbraith et al., 2022), but without integration with other omics. This supports the need for further transcriptome analyses in the RECOVER cohort in the context of a multi-omics approach.
b) **Single-cell transcriptomics:** Bulk transcriptomics measures RNA expression as an average of all cell types present in a sample. This can potentially mask the contribution of rare cell types or cellular states to the transcriptome. Single cell RNA sequencing (scRNAseq) can add further detail to immune phenotyping by measuring the transcriptomes of up to 20,000 individual cells simultaneously. This can provide highly detailed information, albeit at higher cost than bulk transcriptomics. scRNAseq protocols relevant to PASC can include Cellular Indexing of Transcriptomes and Epitopes by Sequencing (CITE-seq) and single cell VDJ sequencing (scVDJ) analyses, which can provide advanced immune phenotyping and T cell receptor/B cell receptor (TCR/BCR) repertoire data, respectively, on the same cell (Cadot et al., 2020; Kim et al., 2020; Lian et al., 2020; Saigusa and Ley, 2020; Frangieh et al., 2021; Mercatelli et al., 2021; Rodahl et al., 2021; Shi et al., 2021; Fan et al., 2022; Xu et al., 2022; He et al., 2022).

#### 3.1.4 Proteomics

a) **Soluble proteins:** Protein-based biomarkers are commonly used for the diagnosis and management of myriad medical conditions and are likely to be useful for the prediction, diagnosis, prognosis and clinical management of PASC. Cytokines, chemokines, antibodies, coagulation factors, growth factors, complement cascade components, peptide hormones, and viral proteins can all be measured by high-content proteomic methods in plasma. Multiple technologies are now available to identify hundreds to thousands of individual proteins from very small volumes of serum, plasma, tissues, or cells, including peripheral blood mononuclear cells (PBMCs). These include mass spectrometry, SOMAscan^®^ assays, Olink^®^ proteomics, and PhIP-seq (phage immunoprecipitation sequencing), to name a few. Furthermore, some of these technologies (e.g., mass spectrometry conjugated with the newest search algorithms such as MSFragger (Kong et al., 2017)) enable the identification of protein isoforms and post-translational modifications, including novel ones. For example, while still under development, the latest SOMAscan^®^ platform measures >7,000 proteins from a mere 125 μL of plasma or serum (Gold et al., 2012). Of critical importance for the study of autoimmunity in PASC, the PhIP-seq technology enables the identification of virtually all auto-antibodies produced by an individual (Mohan et al., 2018). A recent PhIP-seq study in a relatively small cohort identified a common autoreactive pattern in PASC patients and patients who had recovered from acute COVID-19 (Bodansky et al., 2023), raising important questions about the possible role of autoantibodies in PASC.
  Plasma proteomic biosignatures can inform on multiple pathophysiological processes at once, including but not restricted to various forms of inflammation (e.g., systemic, organ-specific, vascular), organ injury, vascular disorders, neurodegeneration, dysregulation of coagulation and fibrinolysis, and remote organ crosstalk *via* the blood. A wealth of proteomics data are already available for acute COVID-19 (Sullivan et al., 2021; Galbraith et al., 2022; Galbraith et al., 2021; D’Alessandro et al., 2020), as well as myriad auto-inflammatory conditions. In the context of an integrated multi-omics strategy, proteomics data could maximize the opportunities to discover mechanisms underlying PASC pathophysiology as well as molecular subtypes, clinically actionable biomarkers and treatment targets.
**b) Cellular proteomics-based immunophenotyping:** Immunophenotyping, which allows for the precise detection of membrane and intracellular proteins using antibodies, has identified signatures that are predictive of subsequent PASC (Peluso et al., 2021). Cellular proteomics-based immunophenotyping technologies can simultaneously quantify, at the single-cell level, expression levels of 40–50 surface and intracellular proteins of immune cells. These include high-parameter flow cytometry (e.g., BD X50), spectral flow cytometry (e.g., Cytek Aurora), and CyTOF (cytometry by time-of-flight, a.k.a. mass cytometry). CyTOF, the most common of these approaches, is a powerful high-dimensional immunophenotyping method that can, in a single specimen, quantify all major subsets of cells using its ∼40 available channels. Alternatively, it can be used to deeply characterize one immune subset of interest (e.g., to interrogate phenotypes, homing properties, effector functions, and self-renewal capacities of T cells) (Neidleman et al., 2021a; Ma et al., 2021; Neidleman et al., 2021b; Neidleman et al., 2020). Phospho-CyTOF also enable analyses of signaling states of individual cells (Bendall et al., 2011). It can also be used to characterize the glycan features of immune cells at the single-cell level, informing on immune functions which are very much modulated by cell-surface glycosylation (Ma et al., 2022). As PASC is multifactorial and heterogeneous, approaches such as CyTOF which allow for broad and specific studies of immune subsets, will be key. Studies can, for example, examine how the global immune landscape is altered during PASC, as well as whether specific subsets implicated in COVID-19 disease progression or PASC (e.g., T cells, myeloid cells, neutrophils) exhibit subset-specific changes that can inform on mechanism of action. Studies that have begun to use CyTOF to explore immunological differences between fully recovered vs individuals with PASC have revealed a dysregulated adaptive immune response in the latter, e.g., global differences in T-cell subsets, sex-specific differences in cytolytic T-cells, increased frequency of T-cells migrating to inflamed tissues but also exhausted T-cells, as well as increased frequency of exhausted T-cells (Yin et al., 2023; Yin et al., 2024). Further studies using larger cohorts are warranted. From a practical standpoint, for the amount of data generated CyTOF is cost-effective and requires relatively few cells relative to if samples were to be analyzed using multiple low-parameter panels implemented in conventional flow cytometry.

#### 3.1.5 Metabolomics

Metabolites are the end products of multiple pathways and often indicate the major phenotype(s) of metabolic and genetic disorders. From diabetes to inborn errors of metabolism, metabolites can often define the key pathways underlying complex diseases and serve as potential biomarkers. Metabolites may also mediate the downstream effects of genomic, epigenomic and transcriptomic processes, and in turn influence these processes to modify PASC phenotypes. As a measure of the status of hundreds of metabolic pathways, the overall metabolome and the lipidome represent biologically and mechanistically informative data streams. The endogenous metabolome captures a broad range of inflammatory processes, energy production, microbial metabolites, organ-specific biomarkers, lipids, carbohydrates, steroids, and amino acids, among other relevant information on physiologic processes. Furthermore, exogenous metabolites capture environmental exposures, including but not restricted to food and supplement intake, toxins (e.g., per-and polyfluoroalkylic substances, also known as PFAS, tobacco byproducts, illicit drugs), and medications (e.g., statins, ibuprofen, selective serotonin reuptake inhibitors), all of which may be important in the development or modification of PASC phenotypes, and cannot be easily predicted by other omics but can potentially impact the results of other omics tests. Importantly, these exogenous metabolites are not measurable by any other mechanisms. Microbial metabolites, also measured by metabolomics assays, may serve as important connectors to microbiome data. The interconnections between the metabolome and other multi-omics profiling illustrates an important aspect of multi-omics strategies: while metabolomics can provide crucial information as a single platform, it acts synergistically with other omics data in elucidating important functional relationships to PASC. Several small studies have demonstrated strong dysregulation of endogenous metabolites associated with particular PASC phenotypes (Valdés et al., 2022). For example, tryptophan metabolism was found to be dysregulated by several groups using metabolomic analyses in blood and urine studies (Bustamante et al., 2022; Dewulf et al., 2022), but the pathogenesis of this phenomenon is unclear. We believe that a comprehensive, longitudinal metabolomics investigation of PASC in the context of a multi-omics strategy in a sufficiently large cohort of patients with deep clinical phenotypes will help define and prioritize functional pathways.

#### 3.1.6 Microbiome

The microbiome has multiple physiological roles in human health, including: i) extracting indigestible ingredients from food and synthesizing nutritional factors; ii) affecting host metabolism; iii) developing systemic and intestinal immunity; vi) providing signals for epithelial renewal and maintaining gut integrity; and iv) secreting anti-microbial products. Alterations of the microbiome may often be an initial disturbance with far-reaching ramifications on disease progression. The gut microbiomes of hospitalized COVID-19 patients were enriched with opportunistic pathogens such as *Clostridium hathewayi*, *Bacteroides nordii*, and *Actinomyces viscosus* (Zuo et al., 2020). In acute COVID-19, the gut microbiome is associated with immune responses and disease severity (Zhang et al., 2021; Maeda et al., 2022; Zuo et al., 2021) and also interacts with the lung microbiome (Zhu et al., 2022). Changes in the gut microbiome could influence respiratory tract infections through the common mucosal immune system. Conversely, respiratory tract dysbiosis and functional disorders due to COVID-19 also affect the digestive tract (Zhu et al., 2022). Studies have demonstrated SARS-CoV-2 interactions with host microbiome/virome communities, clotting/coagulation issues, dysfunctional brainstem/vagus nerve signaling, and immune cells (reviewed in (Proal and VanElzakker, 2021)). There is observational evidence of gut microbiome compositional alterations in patients with long-term complications of COVID-19 (Liu et al., 2022). However, the current studies have sample sizes varying from 8 to 130 patients and few studies followed patients beyond 6 months post-infection (reviewed in (Zhang et al., 2023)). A recent study (Xiong et al., 2023) using multi-omics of microbiome-host interactions identified phenotypic, intestinal microbial, and metabolic biomarkers for short-and long-term myalgic encephalomyelitis/chronic fatigue syndrome. Large amounts of microbiome data can be easily generated at low-cost in the RECOVER adult and pediatric cohorts. These data, when integrated with other multi-omics data, will allow for a better understanding of the virus-microbiome-host interactions and identifying microbial and metabolic biomarkers for PASC. Further studies are also needed to investigate whether microbiota modulation can prevent or facilitate the recovery from PASC.

### 3.2 Considerations on data generation and analysis

#### 3.2.1 Data generation and randomization

Multi-omics data integration can generate valuable knowledge to understand disease pathogenesis. However, multi-omics data can often be burdened by large confounding signals that can prevent accurate modeling and successful discovery. It is therefore essential to appropriately design data collection and generation processes to ensure that such confounders are minimized, and that multi-omics data are amenable to address a large array of important biological questions aiming to characterize, understand and treat PASC. For example, it is usually better to reduce batch effects with a good study design that accurately accounts for them rather than attempting to correct for batch effects after the fact. One successful approach to minimize batch effects involves adequate randomization schemes that minimize risk of contamination of true signals by unwanted variation. Such an approach is powerful when biological questions are defined before data are collected, but often maximizes a distance metric between a measure of interest and the drivers of this unwanted variation at the cost of other potentially meaningful traits. In hypothesis-generating situations, other approaches, such as the inclusion of data generation controls (e.g., reference samples), profiled repeatedly within and across different multi-omics assays, have proven to be an important tool to control for unwanted variation, including confounding from technical variation. However, data generation controls can be complex to define, must contain enough material to be assayed repeatedly, need to capture the full range of biological variation in the multi-omics assessed, and depending on the number needed, can substantially increase data generation cost. These considerations are essential to design a successful multi-omics discovery effort, and it is therefore essential to include data generation experts as well as data scientists who know the biases of each multi-omics profiling technology in teams tasked with designing data generation strategies.

#### 3.2.2 A multi-omics systems-biology approach to data analysis

Each of the omics assays generates vast datasets that require powerful analytical strategies (Gui et al., 2023; Martinez-Bartolome et al., 2018; Lee et al., 2019; Michelhaugh and Januzzi, 2023). Integrating data from multiple omics over time and with clinical, demographic and exposome data is the next level of analytical complexity. A multi-omics approach allows for the integration of multiple layers of information into systems biology models that capture the dynamic interplay between biological processes, allowing not only the study of the functional relationships between the molecular components of PASC, but the elucidation of their causal relationships (Beckmann et al., 2020; Kuijjer et al., 2019; Wang M. et al., 2021; Sonawane et al., 2022; Sonawane et al., 2019; Argelaguet et al., 2020). This approach is critical to understanding the pathogenesis of the clinical manifestations of PASC (Table 3). Integrating multi-omics data with the deep clinical and demographic phenotyping available *via* RECOVER will capture the most complete picture of disease pathophysiology, leading to more accurate identification and characterization of PASC subtypes as compared to individual omics studies of individual patient cohorts (Figure 1).

Multi-omics data are also important in substantiating and validating findings across individual omics platforms (e.g., a genetic polymorphism leading to transcriptomic, proteomic and metabolomics effects). Critical to this is the longitudinal capture of multi-omics data as the clinical presentations of PASC emerge and progress. Given the high-content nature of omics datasets, they support the development of machine learning (ML) class discovery approaches for identification of clinically relevant biosignatures. The rich datasets that will be produced as part of this effort will enable predictive and diagnostic algorithms to identify candidate biomarkers linked to disease outcomes. The thorough integration of these data into meaningful, queryable, and informative models is critical to understand the biological mechanisms, disease subtypes, progression and prognosis of PASC, to investigate the impact of modifiable risk factors and identify potential precision therapeutic approaches to PASC. Inherent in this, is the measure of these data at multiple time points throughout the disease process.

An example of the power of multi-omics approaches is the study of multisystem inflammatory syndrome in children (MIS-C), a serious complication of pediatric COVID-19. Longitudinal plasma bulk transcriptomics, combined with whole blood transcriptomics and plasma DNA epigenomics was recently used to develop multi-organ damage signatures indicative of MIS-C (Loy et al., 2022). This study complements previous genomic, proteomic and immunophenotyping investigations of MIS-C (Sacco et al., 2022; Gruber et al., 2020; Porritt et al., 2021; Carter et al., 2020) to delineate a clearer picture of its pathogenesis. We posit that a single comprehensive, integrated, longitudinal multi-omics approach would have reached similar conclusions as multiple consecutive studies focusing on 1-2 omics each. Such a comprehensive study, performed through centralized labs, would reduce lab-to-lab variability and pre-analytical variability, leveraging a large sample size with rich, highly standardized clinical phenotypes. Furthermore, it is difficult to predict ahead of time which omics would be the most consistent and/or most clinically informative, and which omics data are consistent with each other (e.g., a clinically informative RNA may predict the abundance of an enzyme that produces a metabolite, but if the protein abundance or the metabolite levels are not consistent with RNA abundance, perhaps because of short half-life of the protein or instability of the metabolite, that protein or its metabolite product would not be potential biomarkers or therapeutic targets).Another example of the importance of capturing multi-omics data across demographics and time points is the importance of sex and steroid hormones in the PASC population. Innate and adaptive, humoral and cell-mediated immune responses are impacted by hormones, and their dysregulation contributes to immune-mediated diseases including autoimmunity, a hallmark of PASC (Rojas et al., 2022; Moulton, 2018; Bereshchenko et al., 2018). Ovarian steroids recruit mast cells and T-regs to the uterus during pregnancy (Schumacher et al., 2014). Estradiol causes inflammasome activation in mast cells (Guo et al., 2021). Estradiol deficiency due to menopause and/or hypogonadism contributes to overactivity of the renin-angiotensin-aldosterone system (RAAS), while estrogen can contribute to mast cell activation syndrome (MCAS), which may contribute to the pathogenesis of PASC (O’Donnell et al., 2014; Szukiewicz et al., 2022; Arun et al., 2022). The SARS-CoV2 spike protein binds to and modulates both ACE2 and ERα receptors, and as sex hormones regulate the expression of ACE2 (Solis et al., 2022; Wang H. et al., 2021), the asymmetry in PASC development and clinical presentations between sexes - as well as across menstruation status and menstrual cycle time points - indicates that hormone measurements, which can be performed by metabolomics for non-peptide hormones and by proteomics for peptide hormones, are critical components of a multi-omics strategy.

#### 3.2.3 Task force recommendations

We recommended the following strategy: germline whole genome sequencing (WGS) be performed on every RECOVER participant consented for genetic analysis to be used for GWAS studies. Epigenomics, bulk PBMC transcriptomics, plasma proteomics, plasma targeted metabolomics and stool proteomics should be performed on biospecimens from at least 2 time points per participant (baseline, 90 days and 180 days, or at a minimum baseline and 180 days) on biospecimens from as many participants as possible. Samples taken at later time points during the planned 4-year follow-up period may be analyzed as well in the future, particularly to investigate cases that persist long-term. However, the initial focus should be on the first 180 days post-enrollment, as the number of participants dropping out of the study or being lost to follow-up is likely to increase at later time points. This time window is likely to be long enough to compare COVID-19 cases that do result in PASC to cases that do not, which is one of the primary endpoints of the RECOVER studies, as well as PASC cases that resolve clinically within 180 days from cases that persist beyond that time, while maximizing sample size. Single-cell transcriptomics and/or single-cell immunophenotyping may be performed on subsets of participants from each arm of each cohort, to limit costs. Bioinformatics deconvolution of cellular populations based on bulk transcriptomics should be performed on all available PBMC biospecimens.

It must be pointed out that the adult and pregnant adult RECOVER cohorts include different arms: “acute infected” participants, who enroll within 30 days of a SARS-CoV-2 infection, “post-acute infected”, who enroll after 30 days post-infection, “acute uninfected” enrolled within 30 days of a negative COVID-19 test and “post-acute uninfected”, enrolled after 30 days post-negative test (Horwitz et al., 2023; Reel et al., 2021). This implies that baseline samples taken at enrollment are likely to reflect different pathobiological stages of disease in acute infected *versus* post-acute infected participants. It is also possible that a fraction of the “uninfected” participants will have experienced subclinical infections with SARS-CoV-2. Multi-omics analyses have the potential to identify these cases, particularly through proteomics-based identification of SARS-CoV-2 antibodies not detected by conventional tests. As there are significant differences in study design for the adult and pediatric cohorts (Horwitz et al., 2023; Reel et al., 2021), longitudinal biospecimens will only be available for Tier 2 pediatric participants, while baseline biospecimens will be available for all participants. Also, the amounts of blood/plasma available for the pediatric cohort will depend on the age of participants. With these considerations in mind, maximizing sample size should be the underlying principle. The main objective of this proposed multi-omics analysis is to generate a rich, multidimensional molecular profiling database to match the clinical, pathophysiological and socioeconomics data elements generated by the RECOVER studies. This data should be made available to the scientific community for secondary analyses.

## 4 Conclusion

Based on its analysis of the available evidence, the OMICS Task Force advocated an integrated “big data and systems biology” approach, using multi-omics to analyze biospecimens from the largest possible sample sizes in the RECOVER adult and pediatric cohorts, as opposed to single analyte assays or individual omics in multiple separate studies. This approach will maximize our ability to understand pathogenetic mechanisms in clinically defined patient subgroups, discover PASC molecular subtypes and guide precision therapeutic strategies. Centralized, streamlined omics analyses will limit potential inconsistencies associated with laboratory-to-laboratory and batch variations. In addition, multi-omics assays can capture most clinically assayed biomarkers at cheaper costs. Data generation on this scale can only be accomplished through highly multiplexed approaches, which will maximize opportunities to discover mechanisms underlying PASC pathophysiology.

PASC joins the number of poorly understood, chronic diseases that have been the bane of patients, healthcare providers and clinical researchers. While different clinical presentations of PASC have been described, traditional molecular approaches have thus far failed to produce a deep mechanistic understanding of the etiology, pathogenesis and molecular subtypes of PASC. Many of our recommendations have already been considered by the NIH through the peer-review process, resulting in the creation of a systems biology panel that is currently designing the studies we proposed. Currently, this panel is hammering down the details of the analytical strategies. The NIH RECOVER initiative offers an ideal opportunity to understand PASC in diverse populations, and can serve as a paradigm for the study of other complex, poorly-understood chronic diseases.
